# Plant-Based Diets Are Not Enough? Understanding the Consumption of Plant-Based Meat Alternatives Along Ultra-processed Foods in Different Dietary Patterns in Germany

**DOI:** 10.3389/fnut.2022.852936

**Published:** 2022-04-27

**Authors:** Marlene Ohlau, Achim Spiller, Antje Risius

**Affiliations:** Department of Agricultural Economics and Rural Development, University of Goettingen, Göttingen, Germany

**Keywords:** ultra-processed food, plant-based diet, meat alternatives, dietary pattern, consumption behavior

## Abstract

A low-processive plant-based diet is considered valuable for a sustainable diet profile—it is supposed to meet health as well as environmental concerns. However, there is a growing trend toward plant-based meat alternatives, most of which are to be classified as ultra-processed food (UPF). The paper aimed to understand the consumption of different ultra-processed foods to describe their relation to dietary patterns and sustainability. The objective was (1) to depict the status-quo of consumption of plant-based meat alternatives along with other UPF groups (i.e., convenience products, fast foods, snacks, ultra-processed beverages) in a German sample (*n* = 814) and (2) to investigate the extent to which all examined UPFs are represented in different dietary patterns (vegetarian, flexitarian, regular meat-eaters, high meat-eaters). UPF intake and dietary groups were determined using a food frequency questionnaire (FFQ). Potential factors influencing UPF consumption, such as attitudes toward sustainability and healthy eating practices, were assessed using validated and fitted psychometric scales. Overall, the frequency of UPF consumption varies significantly along the product groups studied. Plant-based meat alternatives were the least consumed food (12.3%), followed by convenience products (57.4%), fast foods (55.9%), ultra-processed beverages (80.1%), and sweet and salty snacks (97.3%). Plant-based meat alternative consumption predominated within a vegetarian diet, while other UPFs, like convenience products, fast foods, sweet and salty snacks, and ultra-processed beverages, were mainly consumed by meat-eaters. Remarkably, flexitarian diets depict low consumption of all types of ultra-processed foods. In order to meet societal sustainability goals, diets and corresponding societal and political actions should emphasize not only plant orientation but also the increase of non- and low-processed foods.

## Introduction

Along with the massive consumption of animal products, high consumption of ultra-processed foods (UPFs) is considered to be one of the main factors for an unsustainable diet, as it is related to both lower dietary quality and intensive food production (1, 2). The market for those foods has rapidly emerged in recent decades worldwide, most notably in high- and middle-income countries. In 2008 UPFs accounted for 46.2% of the total food energy purchased in Germany (3), and although their sales per capita have slightly decreased in Western Europe and North America, they remained the highest by global standards (4). UPFs are formulations of usually many ingredients, most of exclusive industrial use, typically created by a series of industrial techniques and processes. Their main purpose is to create convenient, non-perishable food products that are ready-to-eat or heat, such as frozen, canned, and instant food items (5). And unlike traditional or less processed foods, they are generally energy-dense, high in added sugar, saturated fat, salt, and low in micronutrients and fiber (6). Emerging evidence indicates that UPF consumption is associated with an increased risk of obesity and diet-related non-communicable diseases (e.g., cardiovascular diseases, diabetes, cancers) (3, 7) and, by that, substantially jeopardizing healthy nutrition.

Given today's way of eating threatens society through chronic diseases and damage to Earth's climate, ecosystems, and water resources, a sustainable food system that shifts the global population toward more plant-based foods and less animal-based foods is needed (8–10). Dietary patterns that are predominantly plant-oriented have become a central topic of sustainable development as they benefit the environment and human health (10, 11). Concomitantly, a variety of plant-based innovations have entered the market; alternative protein products that replace those traditionally made from animals. Dairy alternatives (e.g., nut based cheese) as well as meat alternatives in the form of burger patties, sausages, and other meat-like products, that are specifically designed to mimic the taste and experience of eating meat products while being marketed as a healthier and more environmentally friendly alternative (12).

However, these products are industrially produced from largely processed plant-based ingredients and thus should be defined as UPF. Between 2013 and 2018, the number of vegan convenience foods launched worldwide nearly tripled. Fifteen percent of these new product launches in 2018 occurred in Germany alone, the largest share globally (13). Although these meat alternatives may assist in shifting from an animal-based diet to a more plant-based diet, they also have the potential to further fuel UPF consumption and thus re-bounce the effect of the sustainability leverage of the plant-based diets.

The body of evidence on health and environmental assessments of plant-based meat alternatives remains uncertain as yet but is increasingly emerging (14). In regard to health, it was shown that supplementing reduced consumption of red and processed meat with plant-based meat alternatives had a positive effect on the intake of unsaturated fatty acids and dietary fiber, but showed no difference in energy intake and also resulted in lower intake of micronutrients, especially zinc and vitamin B12, and macronutrients such as protein (15, 16). In addition, salt content as well as sodium levels were found to be significantly higher in the plant-based meat alternatives than in the corresponding meat product (17). In contrast, replacing red meat with unprocessed alternative sources of protein such as nuts, legumes, whole grains, and vegetables improves overall diet quality (11, 18) and lowers the risk of diet-related disease (19, 20). When addressing the health effects of plant-based meat alternatives, it is also necessary to consider how and why these products are consumed. UPF is often consumed for convenience and time savings (21–24), which could indeed apply to plant-based meat alternatives. However, this could also impact overall diet quality by losing the necessary skills and knowledge to purchase and cook fresh food (25).

In terms of environmental aspects, plant-based meat alternatives are likely less detrimental to the environment than most meat productions due to refinement losses within the animal production line (14, 26, 27). However, the required extensive processing takes energy and resources, and leads to losses during the transformation from raw material into final products (28, 29). While the overall environmental impact varies along the resources used and the product type of the plant-based meat alternative products (30), several studies ascribe a large life cycle impact to the manufacturing processes (27, 31–33)—sometimes greater than that from cultivation (34). Especially harmful in terms of fossil fuel depletion (35). Critical consideration is warranted that the widely held expectation that such innovations as ultra-processed plant-based protein products are needed to solve the problems of meat leads to a relative neglect of existing alternatives that provide more immediate and significant sustainability gains, particularly legumes (29). Consequently, plant-based meat alternatives could benefit an omnivorous, meat-based diet by substituting for reduced meat consumption, but it is important to be cautious with directly transferring the potential benefits found in previous research on plant-based foods and dietary patterns to processed plant-based meat alternatives.

This paper extends previous research approaches to UPF consumption by including plant-based meat alternatives to provide comprehensive and, more importantly, contemporary information on consumption behavior. Furthermore, it aims to determine which factors are associated with UPF consumption. Other studies on UPF have already identified some influencing factors. Among the first was Candel (36), who found that convenience food consumption was negatively related to enjoyment of cooking, engagement with food, and seeking variety. Verlegh and Candel (37) found that sociability is another variable that seems to be related to the consumption of convenience foods. They examined the influence of social situations, such as eating alone or eating with family or friends. Following this, Brunner et al. (24) found a positive correlation with low cooking skills for the first time. In addition, several studies unveiled the impact of socio-demographic characteristics, with females, multi-person households, having children, and higher education level negatively correlated with its consumption, while younger age mostly positively correlates [e.g., (21, 24, 38–41)].

However, the goal of the current research was to expand this line of research by identifying new drivers due to the new product group, i.e., plant-based meat alternatives. Motives that go beyond the consumption of conventional UPF by following those of sustainable food choices, such as attitude toward meat consumption and healthy nutrition, moral and ethical beliefs, animal welfare concerns, environmental impact of food, and sensory considerations [e.g., (42–45)]. All of these drivers could likewise apply to the consumption of meat alternatives. In addition, a certain curiosity for new products or willingness to try novel or unfamiliar foods could be substantial (46).

This study aims (1) to depict the status quo of consumption of plant-based meat alternatives together with other UPF groups (i.e., convenience foods, fast foods, sweet and salty snacks, ultra-processed beverages) in Germany, (2) to determine the extent to which all examined UPFs are represented in different dietary pattern, both omnivore- and plant-oriented, and (3) to investigate factors associated to UPF consumption (including sociodemographic characteristics, attitudes, and behaviors referring to healthy eating, cooking, food choices, purchase places, and meat attachment).

Considering the continuously high or in some parts of the world even growing share of UPF in the food market, including the upcoming market for ultra-processed plant-based meat alternatives, the present study contributes to a better understanding and evaluation of the quality of different dietary patterns and thus, in the long term, to the support of food policies, dietary guidelines, and labeling regulations that educate consumers on sustainable and healthy food choices.

## Materials and Methods

### Design and Study Sample

This study is based on data collected in a quantitative online survey carried out in May and June 2019 in Germany. The online questionnaire was generated using the online survey software Unipark (Tivian XI GmbH, Germany) and consisted of various sections, including sociodemographic, food frequency questionnaire (FFQ), dietary habits, food-related attitudes and behaviors focusing meat consumption and sustainability, and health conditions. Items were randomized within a question battery. The study was part of an interdisciplinary project supervised by the Ethics Committee of the University of Goettingen, to which the rules of scientific practice of the Declaration of Helsinki apply. The Ethics Committee granted ethical approval for the study.

Participants were invited to the survey via the market research company respondi AG. A soft launch was carried out with 97 participants to assess and adjust the feasibility. Quotas were set for gender, age, and education based on the German population. A total of 2,347 respondents took part in the online survey. Out of these, 284 respondents were rejected due to quota setting, 786 respondents were screened out due to lack of attention/thoroughness (quality checks, randomly positioned within the items; e.g., “Please click on “strongly disagree”), and 257 respondents did not complete the survey. Thus, an initial data set with 1,020 respondents could be generated. In order to ensure data quality, the data set was adjusted for subjects who responded too fast (i.e., speeder; threshold of one-third of the median survey time was determined within pre-testing), stereotypically (e.g., straight-liners, noticeably identical answers within several item batteries), or inconsistently (e.g., meat consumption within self-declared vegetarians) response behavior (*n* = 206). After data cleaning, the final sample contains the data of 814 respondents. Due to the reduction of the sample size, there are small deviations from the official population statistics in Germany, with females being slightly overrepresented while males being slightly underrepresented. Middle-aged people between 25 and 64 years are overrepresented, while younger people under 24 and people aged 65 and above are slightly underrepresented. The share of middle and high educated people regarding school education is lower than the overall German population. Detailed characteristics are shown in Table 1.

**Table 1:** Sample characteristics.

**Variable**	**Sample (*****N*** **=** **814)**		Population (Germany)*
	***n***	**%**	**%**
**Gender**			
Male	358	44.0	49.3
Female	455	55.9	50.7
Diverse	1	0.1	–
**Age (in years)**			
18–24	76	9.4	7.4
25–39	191	23.5	19.0
40–64	397	48.8	35.1
65+	150	18.3	22.0
**Education**			
Low	286	35.1	35.0
Middle	220	27.0	30.0
High	221	27.1	33.5

* *Based on: “DESTATIS”: www.destatis.de*.

### Measures

#### UPF Consumption

UPFs were classified into the following groups: Plant-based meat alternatives, convenience foods, fast foods, sweet and salty snacks, and ultra-processed beverages (Table 2). In order to distinguish non-processed plant-based proteins, such as legumes, or less processed plant-based meat alternatives, such as tofu and tempeh, from ultra-processed meat imitations (e.g., vegetarian meat and sausages), these were asked in advance as part of the questionnaire.

**Table 2:** Classification of ultra-processed foods.

**UPF group**	**Items**
Plant-based alternatives to meat and meat products	Plant-based meat sauce (e.g., bolognese style), vegetarian sausages, vegan sausages, vegetarian meat (whole cuts), vegan meat (whole cuts), vegetarian meat (cold cuts), vegan meat (cold cuts), plant-based cheese
Convenience foods (ready-to-heat)	Canned soup and stews, instant sauces and soups, chicken nuggets, pizza, canned noodle dishes (e.g., ravioli), frozen potato dishes (e.g., pommes), frozen noodle dishes, frozen prepared breads (e.g., garlic baguette), frozen fish dishes (e.g., fish sticks)
Fast foods (ready-to-eat)	Burger, baguettes and sandwiches, pommes, pizza, sausages, falafel
Sweet and salty snacks	Chips, crackers, pretzels, cakes, cookies, chocolate, gummy bears, ice cream, candies
Ultra-processed beverages	Lemonades, cola, ice-tea, carbonized water-juice mixes, energy drinks, sports drinks

Further, in order to give respondents an accurate understanding of which products were meant with respect to all product categories (i.e., plant-based meat alternatives, convenience foods, fast foods, sweet and salty snacks, and ultra-processed beverages), definitions were first provided in the questions (e.g., “In the following, we would like to know more about your consumption of plant-based meat alternatives. This refers to alternative sources of protein that aim to imitate meat, i.e., that have similarities to products of animal origin, such as vegetarian/vegan cold cuts, schnitzel, burger patties, sausages, and other vegetarian/vegan products.”). All products were then listed, which can be found in Table 2.

Subsequently, participants were asked about the frequency of consumption of each food group (displaying the included products) in the last 4 weeks. Responses were on a 9-point scale: “not once,” “1 time per month,” “2-3 times per month,” “1-2 times per week,” “3-4 times per week,” “5-6 times per week,” “1 time per day,” “2-3 times per day,” “>3 times per day.” The last three scale points were then merged to “1 or more times per day”, resulting in a 7-point scale.

#### Dietary Identity

According to their meat consumption, participants were divided into four dietary groups: vegetarians, flexitarians, regular meat-eaters, and high meat-eaters. Participants were categorized as vegetarians if they self-identified as such (including vegan, lacto-vegetarian, ovo-vegetarian, ovo-lacto-vegetarian, or pesco-ovo-lacto-vegetarian) and if they had not consumed meat in the past 4 weeks. The categorization of meat-eaters was based on information about the frequency of meat consumption, which was derived from the FFQ. That included processed and unprocessed meat products and was calculated by summing the meat intake of each meat product by frequency category to estimate daily intake in terms of servings. According to the FAO definition^1^, the consumption of one serving of meat per week or less was classified as flexitarian. A further distinction was made between regular meat-eaters (i.e., “more than one serving per week, up to one serving per day”) and high meat-eaters (i.e., “several times per day”).

#### Determinants of UPF Consumption

Fifty-one items within nine constructs were considered to gain better insight into the impacts of UPF intake. The constructs related to cooking behavior, dietary habits, and consumption habits. Whenever possible, existing scales were used; otherwise, scales were adapted to maintain comparability along with constructs. Most item scales had good internal reliability (Cronbach's alpha). Two scales yielded a rather low value: Conventional grocery stores (i.e., delivery services, supermarket, discounter) (three items, α = 0.13) and food choice motives (other) (i.e., taste, price, freshness) (five items, α = 0.53) and were not included in the analyses (Supplementary Material Table A1). Constructs related to sociability did not meet prespecified criteria, either through the principal component analysis applied or in terms of scale reliability, and thus were not included in the analysis either. Additionally, socio-demographic characteristics were added as predicting variables.

##### Cooking Behavior

Participants rated seven items about their cooking behavior. First, it was asked how often the participant cooks a meal for him/herself. Seven response options were available for this question: never, only for special occasions, less than once a week, 1 or 2 days a week, some days (3–4 a week), most days (5–6 a week), and every day. The scale was derived from Adams et al. (47) and edited afterward to a 5-point scale from “never” to “very often.” Second, it was asked the degree of processing of the ingredients used for cooking (e.g., “I use fresh and unprocessed ingredients,” “I use ready-made products for cooking”) on a 5-point scale from “never” to “very often,” also based on and altered from Adams et al. (47). Then it was asked how much time the participant spent cooking on weekdays and weekends with time intervals ranging from: <15 min, between 16 and 30 min, between 31 and 45 min, between 46 and 60 min, more than 60 min. This question was derived from Ducrot et al. (48) and adapted to a 5-point scale. Exploratory factor analysis showed that the items were explained by two factors. The first factor related to items on a lower frequency of cooking, less time spent on cooking, and a higher degree of processing of the ingredients used. The second factor, in contrast, referred to the items that captured a higher time commitment to cooking and less processed food. The two factors explained 59.3% of the item variance. The items of the first factor indicating less frequent cooking with mainly instant ingredients had reliability of α = 0.74. The items of the second factor indicating higher engagement in cooking (e.g., more often, using fresh ingredients) had reliability of α = 0.76.

##### Meat Attachment

Nine items on attitudes toward meat consumption were included that based on the meat attachment questionnaire by Graça et al. (49) (e.g., “It is a pleasure to eat meat because it is part of many good-tasting dishes”). Two items were added that follow the concept of “vegaphobia” by Vandermoere et al. (50), first mentioned by Cole and Morgen (51), which describes a negative attitude toward a vegan diet (e.g., “I find people who eat vegan weird”). Item agreement was assessed on a 5-point Likert scale ranging from “strongly disagree” to “strongly agree.” The items were captured by one factor that explained 49.7% of the variance in the items. Cronbach's alpha was high (α = 0.90).

##### Food Innovativeness

The questionnaire included four items on food innovativeness (e.g., “I like to try new food trends,” “I like the food of other cultures”), based on the food-related lifestyle scale by Scholderer et al. (52). The items were answered on a 5-point Likert scale from “strongly disagree” to “strongly agree.” The three items were captured by one factor explaining 62.9% of the item variance and had a reliability of α = 0.74.

##### Dietary Guidelines

Eight items for personal consideration of dietary guidelines, based on recommendations of the German Society for Nutrition^2^ (e.g., “I make sure to drink enough water”) were included in the questionnaire. Items were answered on a 5-point Likert scale ranging from “strongly disagree” to “strongly agree.” The items were captured by one factor explaining 44.8% of the item variance. Cronbach's alpha was high (α = 0.82).

##### Food Choices Motives

Fourteen items on food choice motives were included using a modified food choice questionnaire (FCQ) based on the scale developed by Steptoe et al. (53) and expanded by Verain et al. (42). Further items were developed by discussing, adjusting, and reflecting current motivations for food choice motives. Respondents were asked to indicate on a scale of 1 (“not at all important”) to 5 (“very important”) to what extent the motives were decisive for the purchase of a food product. The principal component analysis identifies two underlying factors with items explaining 61.0% of the item variance: one consists of food characteristics related to sustainability (i.e., organic production, regionality, animal welfare, animal husbandry, seasonality, label, transport, naturalness, fair trade, non-GMO, DLG quality testing (i.e., by German Agricultural Society), whereas the second factor corresponds to price, taste, and freshness. However, as Cronbach's alpha of the letter was low (α = 0.53), and only the sustainability items formed a reliable scale (α = 0.95), only these items were included in the analysis.

##### Food Shopping Locations

Seven items on consumption behavior were included in the questionnaire regarding where to buy food (e.g., organic food store, supermarket, farmers market) partly based on Zepeda and Nie (54), Grunert (55), Korn and Hamm (56), and (54). The items were answered on a 5-point scale from “never” to “very often.” Items were captured on two factors that explained 47.2% of the item variance. The first factor was related to organic and regional food purchases; the second factor was related to delivery services, supermarkets, and discounters. The first factor showed reliability of α = 0.75, while the second factor had an insufficient alpha of α = 0.13 and was therefore excluded from the analysis.

##### Sociodemographic and Lifestyle Factors

Socioeconomic and demographic variables were included related to gender, age, and education. Educational level was divided into low, middle and high based on the categories: No education, certificate of secondary education, general qualification for university entrance, university degree.

### Data Analysis

All analyses were performed with the statistical software package IBM SPSS Statistics version 27.0 (IBM Corp., Somers, NY, USA.). Differences between sociodemographic and health characteristics of vegetarians, flexitarians, regular and high meat-eaters were tested using Chi square with Cramer's V or one-way ANOVA with eta squared (η^2^) to estimate effect size. The consumption frequencies of plant-based meat alternatives, convenience products, and fast-food products, previously measured on a 7-point categorical scale, were nominally divided into consumer and non-consumer groups, due to the low distribution of responses in the upper frequency categories (from 1 to 2 times per week upwards). For sweet and salty snacks and ultra-processed beverages, the original 7-point categorical scale was retained. Consumption frequencies were calculated using cross-tabulations and chi-square with Cramer's V to examine differences between the dietary groups regarding their consumption behavior.

Exploratory factor analysis of principal components with varimax rotation was conducted to examine unidimensionality of the constructs regarding cooking behavior, eating habits (i.e., sociability, meat attachment, food innovativeness, dietary guidelines), and consumption behavior (i.e., food choice motives, food shopping locations), to determine the common variance among the items, and to identify the factors or dimensions underlying the data (see Supplementary Material Table A1). The constructs were presumed to meet the KMO criterion of at least 0.6 (57, 58). Cronbach's alpha scores of the items belonging to each factor were calculated, to assess the internal consistency of the constructs. Factors were considered reliable when Cronbach's alpha coefficient was above the lower limit of 0.6.

Hierarchical logistic regression models were used to identify associations with binary, and ordinal outcomes, respectively. The variables were entered into the model in blocks to investigate the extent to which factors predict the consumption behavior: First, the unadjusted relationship between dietary pattern and consumption behavior was tested (see Supplementary Material Tables A2–A6). Then, the models were adjusted by adding sociodemographic variables (gender, age, education), followed by attitudinal and behavioral factors. A *p*-value <0.05 indicated statistical significance.

## Results

### Characteristics of Dietary Groups

As shown in Table 3, the number of vegetarians was low at 7.9% of the total sample, whereas regular and high meat eaters made up the largest subsample. The descriptive characteristics of the dietary groups show that in terms of gender, the proportion of women decreased as meat consumption increased. Thus, vegetarians were more often women (60.9%), while high meat-eaters were more often men (54.6%). There were also significant differences with respect to age and education level. Within vegetarians, the average age was lower than compared to frequent meat-eaters. In addition, within a low level of education, there were fewer vegetarians and regular meat eaters than high meat-eaters. For this reason, gender, age, and level of education are used as covariates in further analyses. Regarding household composition (number of persons and children), region, income, and health data, there was no significant difference between the dietary groups.

**Table 3:** Characteristics according to dietary groups [vegetarians (no meat), flexitarians (≤1 × meat/week), regular eat-eaters (≤7 × meat/week), high meat-eaters (>1 × meat/ day)].

	**Vegetarians**	**Flexitarians**	**Regular meat-eaters**	**High meat-eaters**	**x^**2**^ or F**
Number of the sample	*64*	*192*	*285*	*273*	
Percentage of the sample	7.9	23.6	35.0	33.5	
**Gender (%)**					
Female	60.9%^a,b^	65.6%^a^	58.2%^a^	45.4%^b^	*x^2^ =* 21.315, V = 0.162***
Mean Age (SD)	27.8^a^	32.7^a,b^	32.7^a,b^	34.3^b^	F = 3.101, η^2^ = 0.107*
**Level of education (%)**					
Lower	26.6%^a,b^	34.9%^a,b^	30.9%^a^	41.8%^b^	*x^2^ =* 9.590, V = 0.109*
Middle	28.1%	27.1%	31.2%	22.3%	*x^2^ =* 5.625, V = 0.083
Higher	35.9%	28.1%	24.6%	27.1%	*x^2^ =* 3.557, V = 0.066
Mean number of persons in the household (SD)	2.0	2.1	2.1	1.3	F = 2.594, η^2^ = 0.098
Mean number of children (SD)	1.3	1.2	1.3	1.4	F = 1.211, η^2^ = 0.067
**Region of residence (%)**					
Rural area	31.3%	17.2%	18.6%	22.7%	*x^2^* = 7.237, V = 0.094
Small town	12.5%	24.5%	16.1%	21.2%	*x^2^* = 7.599 V = 0.097
Middle-sized town	21.9%	27.1%	31.9%	23.8%	*x^2^ =* 5.711, V = 0.084
Major city	34.4%	31.3%	33.3%	32.2%	*x^2^* = 0.337 V = 0.020
**Household income (%)**					
Below 1,200€	31.1%	25.5%	22.5%	19.4%	*x^2^* = 5.192, V = 0.080
>1,200€−2,400€	37.5%	39.1%	35.1%	33.7%	*x^2^* = 1.557, V = 0.044
>2,400€−3,600€	18.8%	24.5%	24.9%	29.3%	*x^2^* = 3.705., V = 0.067
>3,600€−4,800€	6.3%	5.7%	10.9%	12.5%	*x^2^* = 7.043, V = 0.093
Above 4,800€	6.3%	5.2%	6.7%	5.1%	*x^2^* = 0.766, V = 0.031
Mean BMI* (SD)	27.1 (8.092)	28.6 (9.115)	27.7 (7.576)	27.6 (8.617)	F = 1.761, η^2^ = 0.081
**High level of physical activity (%)**					
<2 h/week	75.8%	70.1%	76.4%	73.5%	*x^2^* = 2.504, V = 0.056
≥2 h/week	24.2%	29.9%	23.6%	26.5%	
**Low level of physical activity (%)**					
<2 h/week	34.9%	39.3%	36.4%	41.2%	*x^2^* = 1.714, V = 0.046
≥2 h/week	65.1%	60.7%	63.6%	58.8%	

^*^BMI (Body Mass Index): <18.5 = underweight; 18.5–24.9 = normal weight; 25.0–30.0 = overweight; >30.0 = obese. Percentages and means with different superscript letters (

a,b *) differ significantly (Bonferroni test, p < 0.05)*.

* *p < 0.05;*

** *p < 0.01;*

*** *p < 0.001*.

### Plant-Based Meat Alternatives, Convenience Products, and Fast Foods

Table 4 shows the consumption of plant-based meat alternatives, convenience foods, and fast foods by dietary patterns. Overall, 12.3% of respondents (*n* = 100) have consumed plant-based meat alternatives in the past month, with a frequency of consumption predominantly ranging from 1 time per month to 1–2 times per week. Significantly more consumers of such products were vegetarians. Of these, 39.1% reported consuming meat alternatives at least once a month. In contrast, only about 10% of meat eaters said they consumed such products, with no difference between low, regular, and high meat-eaters.

**Table 4:** Consumption of plant-based meat alternatives, convenience products, and fast foods among dietary groups [vegetarians (no meat), flexitarians (≤ 1 × meat/week), regular eat-eaters (≤7 × meat/week), high meat-eaters (>1 × meat/day)].

	***n***	**Vegetarians** ***n* = 64**	**Flexitarians** ***n* = 192**	**Regular meat-eaters** ***n* = 285**	**High meat-eaters** ***n* = 273**	***x*^2^ and V/*p***
**Plant-based meat alternatives**						
Non-consumers	713	60.0%^a^	89.6%^b^	88.4%^b^	91.9%^b^	*x*^2^= 47.800; V = 0.242***
Consumers	100	39.1%^a^	10.4%^b^	11.6%^b^	8.1%^b^	
1 time per month	37	9.4%	5.2%	3.9%	3.7%	n.s.
2–3 times per month	28	12.5%^a^	3.1%^b^	2.1%^b^	2.9%^b^	0.001
1–2 times per week	25	7.8%^a^	1.6%^b^	4.9%^b^	1.1%^b^	0.004
3–4 times per week	0	–	–	–	–	n.s.
5–6 times per week	2	3.1%	–	–	–	0.000
1 or more times per day	8	6.3%^a^	0.5%^b^	0.7%^b^	0.4%^b^	0.000
**Convenience products**						
Non-consumers	345	53.1%^a,b^	47.6%^a^	43.8%^a,b^	35.3%^b^	*x*^2^= 10.996; V = 0.117*
Consumers	465	46.9%^a,b^	52.4%^a^	56.2%^a,b^	64.7%^b^	
1 time per month	139	18.8%	17.8%	15.2%	18.4%	n.s.
2–3 times per month	189	15.6%	19.4%	23.7%	27.6%	n.s.
1–2 times per week	105	7.8%	13.1%	12.4%	14.7%	n.s.
3–4 times per week	24	1.6%	1.6%	3.5%	3.7%	n.s.
5–6 times per week	7	3.1%	0.5%	1.4%	0.0%	n.s.
1 or more times per day	1	–	–	–	0.4%	n.s.
**Fast foods**						
Non-consumers	358	57.8%^a^	50.5%^a,b^	41.9%^a,b^	38.7%^b^	*x*^2^= 11.798; V = 0.121**
Consumers	453	42.2%^a^	49.5%^a,b^	58.1%%^a,b^	61.3%^b^	
1 time per month	144	18.8%	18.2%	19.7%	15.1%	n.s.
2–3 times per month	198	15.6%^a^	19.3%^a,b^	24.3%^a,b^	30.3%^b^	0.015
1–2 times per week	82	3.1%	9.9%	9.9%	12.2%	n.s.
3–4 times per week	21	1.6%	2.1%	2.8%	3.0%	n.s.
5–6 times per week	7	3.1%	–	1.4%	0.4%	n.s.
1 or more times per day	1	–	–	–	0.1%	n.s.

*Percentages with different superscript letters (^a,b^) differ significantly (Bonferroni test, p < 0.05)*.

* *p < 0.05;*

** *p < 0.01;*

*** *p < 0.001*.

57.4% of all respondents (*n* = 345) reported having consumed convenience foods in the last month, with consumption significantly higher among regular meat-eaters than flexitarians. Regarding the consumption of ultra-processed fast food products, 55.9% of respondents (*n* = 358) stated that they had consumed such products in the last 4 weeks, with significantly higher consumption within the high meat-eaters than vegetarians.

### Sweet and Salty Snacks and Ultra-processed Beverages

Table 5 shows the consumption of sweet and salty snacks and ultra-processed beverages, according to dietary patterns. Overall, 97.3% of respondents reported consuming sweet and salty snacks within the last 4 weeks, with 40.7% of participants stating to consume snacks three times and more per week. Consumption is significantly higher among regular meat-eaters than flexitarians within the category “3–4 times per week.” Regarding the consumption frequency of ultra-high-processed beverages, 80.1% of respondents stated that they had consumed such beverages within the last 4 weeks, with 32.7% reporting daily consumption. In terms of dietary habits, consumption differs in the frequency of “not once” between vegetarians and regular meat-eaters and “once a day” between flexitarians and high meat-eaters.

**Table 5:** Consumption of sweet and salty snacks and ultra-processed beverages among dietary groups [vegetarians (no meat), flexitarians (≤ 1 × meat/week), regular eat-eaters (≤ 7 × meat/week), high meat-eaters (>1 × meat/day)].

	***n***	**Vegetarians (*n* = 64)**	**Flexitarians (*n* = 192)**	**Regular meat-eaters (*n* = 285)**	**High meat-eaters (*n* = 273)**	***p***
**Sweet and salty snacks**						
Not once	22	6.3%	3.1%	3.2%	1.1%	n.s.
1 time per month	71	10.9%	11.5%	8.1%	7.0%	n.s.
2–3 times per month	161	23.4%	23.4%	18.2%	18.0%	n.s.
1–2 times per week	228	23.4%	27.1%	29.8%	27.9%	n.s.
3–4 times per week	185	14.1% ^a,b^	17.7% ^a^	26.3% ^a,b^	24.6% ^b^	0.043
5–6 times per week	107	17.2%	11.5%	10.2%	16.5%	n.s.
1 or more times per day	39	4.7%	5.7%	4.2%	4.8%	n.s.
**Ultra-processed beverages**						
Not once	162	40.6%^a^	20.3%^b^	19.6%^b^	15.0%^b^	0.000
1 time per month	44	3.1%	6.8%	5.3%	5.1%	n.s.
2-3 times per month	96	9.4%	13.5%	12.3%	10.6%	n.s.
1-2 times per week	125	14.1%	17.2%	13.0%	16.8%	n.s.
3-4 times per week	75	4.7%	10.4%	9.8%	8.8%	n.s.
5-6 times per week	46	4.7%	6.8%	3.9%	7.0%	n.s.
1 or more times per day	266	23.4%^a,b^	25.0%^a^	36.1%^b^	32.7%^a,b^	0.011

*Percentages with different superscript letters (^a,b^) differ significantly (Bonferroni test, p < 0.05)*.

### Predictors of UPF Consumption

Regression analysis for consumption of plant-based meat alternatives shows that a vegetarian diet significantly predicted plant-based meat alternative product consumption (see Table 6). In addition, gender, age, and education significantly influenced consumption behavior. Females, increasing age, and higher education were more likely associated with plant-based meat alternative consumption. Regarding attitudinal and behavioral variables, which were included subsequently, low cooking frequency and practice, innovativeness, meat attachment, sustainability-related food choice motives, attitude toward a healthy diet, and shopping in organic markets emerged as significant predictors. Meat attachment and sustainability-related food choice motives had a negative effect on consumption. The impact of dietary patterns and sociodemographic variables, with the exception of age, diminished after adjusting the model for attitudinal and behavioral variables.

**Table 6:** Results of a binary logistic regression analysis predicting consumption of plant-based meat alternatives (*N* = 814).

	***B* ^a^**	**SE *B***	**OR**	***B* ^b^**	**SE *B***	**OR**
**Diet**						
Vegetarian	**2.008**	**0.414**	7.445***	0.248	0.565	1.282
Flexitarian	0.459	0.386	1.582	0.189	0.442	1.208
Regular meat-eater	0.456	0.363	1.582	0.372	0.405	1.450
High meat-eater (reference)						
**Gender**						
Male	**−0.668**	**0.288**	0.513*	−0.336	0.335	0.715
**Female (reference)**						
*Age (years, continuous)*	**−0.029**	**0.010**	0.971**	**−0.037**	**0.012**	0.963**
**Education**
Low	**−0.797**	**0.367**	0.451*	−0.108	0.433	0.897
Middle	−0.278	0.298	0.757	0.405	0.348	1.499
High (reference)						
**Attitudinal and behavioral variables (factors, continuous)**				**0.376**	**0.163**	1.457*
Infrequent cooking/prepared ingredients						
Frequent cooking/fresh ingredients				−0.227	0.176	0.797
Meat attachment				**−1.191**	**0.204**	0.304***
Food innovativeness				**0.475**	**0.168**	1.608**
Dietary guidelines				**0.416**	**0.194**	1.515*
Sustainable food choice motives				**−0.406**	**0.193**	0.666*
Organic food stores				**0.562**	**0.155**	1.753***
*Nagelkerke R square (%)*	19.0			42.0		

*Significant predictors are displayed in bold font.*

*B, Beta coefficient; SE, Standard Error; OR, Odds Ratio.*

a *Adjusted for gender, age, and education.*

b *Adjusted for gender, age, education, attitudinal and behavioral variables.*

* *p < 0.05;*

** *p < 0.01;*

*** *p < 0.001*.

Table 7 shows that convenience food consumption was not predicted by dietary pattern but was statistically significantly higher the lower the age. There was also a significant relationship between consumption and cooking behavior. Those who cooked less often, spent less time cooking, and used ready-processed ingredients were more likely to consume higher amounts of convenience foods.

**Table 7:** Results of a binary logistic regression analysis predicting convenience food consumption (*N* = 814).

	***B^a^***	**SE *B***	**OR**	***B^b^***	**SE *B***	**OR**
**Diet**						
Vegetarian	−0.512	0.325	0.599	−0.324	0.407	0.723
Flexitarian	−0.395	0.224	0.673	−0.278	0.248	0.757
Regular meat-eater	−0.259	0.208	0.772	−0.094	0.227	0.910
High meat-eater (reference)						
**Gender**						
Male	−0.117	0.172	0.890	−0.199	0.196	0.820
Female (reference)						
*Age (years, continuous)*	**−0.023**	**0.006**	0.978***	−0.008	0.007	0.992
**Education**						
Low	0.196	0.221	1.217	0.075	0.250	1.078
Middle	−0.006	0.216	0.994	−0.068	0.240	0.934
High (reference)						
**Attitudinal and behavioral variables (factors, continuous)**						
Infrequent cooking/prepared ingredients				**0.950**	**0.114**	2.585***
Frequent cooking/fresh ingredients				0.010	0.101	1.011
Meat attachment				−0.128	0.112	0.880
Food innovativeness				0.019	0.097	1.020
Dietary guidelines				−0.107	0.117	0.898
Sustainable food choice motives				−0.058	0.110	0.944
Organic food stores				0.143	0.106	1.154
*Nagelkerke R square (%)*	4.0			22.9		

*Significant predictors are displayed in bold font.*

*B, Beta coefficient; SE, Standard Error; OR, Odds Ratio.*

a *Adjusted for gender, age, and education.*

b *Adjusted for gender, age, education, attitudinal and behavioral variables.*

* *p < 0.05;*

** *p < 0.01;*

*** *p < 0.001*.

Consumption of fast foods was statistically significantly negatively associated with a vegetarian diet (Table 8). Likewise, fast food consumption was significantly higher with decreasing age. After adjusting the model for attitudinal and behavioral variables, there was a significant relationship between consumption and low cooking frequency and practice and a relationship to shopping in food stores related to organic and regional food.

**Table 8:** Results of a binary logistic regression analysis predicting fast food consumption (*N* = 814).

	***B* ^a^**	**SE *B***	**OR**	***B* ^b^**	**SE *B***	**OR**
**Diet**						
Vegetarian	**−0.889**	**0.345**	0.411**	**−0.961**	**0.401**	0.383*
Flexitarian	−0.397	0.236	0.672	−0.374	0.249	0.688
Regular meat-eater	0.000	0.219	1.000	0.068	0.227	1.071
High meat-eater (reference)						
**Gender**						
Male	0.206	0.182	1.229	0.175	0.197	1.191
Female (reference)						
*Age (years, continuous)*	**−0.050**	**0.007**	0.951***	**−0.044**	**0.007**	0.957***
**Education**						
Low	−0.342	0.230	0.710	−0.263	0.246	0.769
Middle	−0.131	0.233	0.877	−0.076	0.244	0.927
High (reference)						
**Attitudinal and behavioral variables (factors, continuous)**						
Infrequent cooking/prepared ingredients				**0.356**	**0.099**	1.427***
Frequent cooking/fresh ingredients				−0.111	0.101	0.895
Meat attachment				−0.081	0.113	0.922
Food innovativeness				0.137	0.097	1.146
Dietary guidelines				−0.082	0.115	0.921
Sustainable food choice motives				−0.111	0.110	0.895
Organic food stores				**0.277**	**0.107**	1.319**
*Nagelkerke R square (%)*	18.7			23.1		

*Significant predictors are displayed in bold font.*

*B, Beta coefficient; SE, Standard Error; OR, Odds Ratio.*

a *Adjusted for gender, age, and education.*

b *Adjusted for gender, age, education, attitudinal and behavioral variables.*

* *p < 0.05;*

** *p < 0.01;*

*** *p < 0.001*.

Table 9 shows that adopting a vegetarian and flexitarian diet statistically significantly decreased the likelihood of snack consumption. It further revealed that the female gender and younger age were significantly related to consumption. When the model was adjusted for attitude and behavior, it shows that low cooking frequency and little cooking practice was significant for snack consumption and the likelihood of consumption increases with decreasing consideration of dietary guidelines for healthy eating. The relationship to dietary pattern and sociodemographic variables, with the exception of age, decreased.

**Table 9:** Results of an ordinal regression analysis predicting sweet and salty snack consumption (*N* = 814).

	**Est^a^**	**SE**	**OR**	**Est^b^**	**SE**	**OR**
**Diet**						
Vegetarian	**−0.699**	**0.263**	0.497**	−0.439	0.323	0.645
Flexitarian	**−0.531**	**0.179**	0.588**	−0.360	0.199	0.698
Regular meat-eater	−0.258	0.162	0.773	−0.141	0.180	0.869
High meat-eater (reference)						
**Gender**						
Male	**−0.267**	**0.136**	0.766*	**−0.379**	**0.157**	0.685*
Female (reference)						
*Age (years, continuous)*	**−0.016**	**0.005**	0.984***	−0.005	0.006	0.995
**Education**						
Low	0.005	0.175	1.005	−0.224	0.198	0.799
Middle	−0.207	0.171	0.813	−0.252	0.191	0.777
High (reference)						
**Attitudinal and behavioral variables (factors, continuous)**						
Infrequent cooking/prepared ingredients				**0.244**	**0.078**	1.276**
Frequent cooking/fresh ingredients				0.132	0.081	1.141
Meat attachment				0.010	0.089	1.010
Food innovativeness				0.116	0.078	1.123
Dietary guidelines				**−0.386**	**0.093**	0.680***
Sustainable food choice motives				0.089	0.088	1.093
Organic food stores				0.081	0.084	1.084
*Nagelkerke R square (%)*	3.6			8.9		

*Significant predictors are displayed in bold font.*

*Est, Estimate; SE, Standard Error; OR, Odds Ratio.*

a *djusted for gender, age, and education.*

b *Adjusted for gender, age, education, attitudinal and behavioral variables.*

* *p < 0.05;*

** *p < 0.01;*

*** *p < 0.001*.

It has been shown that there is a relationship between ultra-high-processed beverages and plant-based diets, as the likelihood of consumption decreases significantly with adopting a vegetarian diet (Table 10). Regarding socio-demographic variables, it becomes apparent that men were more likely to consume those beverages than women. A positive attitude toward meat consumption and sustainable food choice motives appeared to be statistically significantly related to the consumption after adding attitudinal and behavioral variables. Also, those who pay attention to a healthy diet were more likely to consume lower amounts of ultra-processed beverages. The impact of dietary pattern and gender decreased after adjusting the model for attitudinal and behavioral variables.

**Table 10:** Results of an ordinal regression analysis predicting ultra-processed beverage consumption (*N* = 814).

	**Est^a^**	**SE**	**OR**	**Est^b^**	**SE**	**OR**
**Diet**						
Vegetarian	**−0.883**	**0.264**	0.414***	−0.268	0.329	0.765
Flexitarian	−0.278	0.179	0.757	−0.076	0.200	0.927
Regular meat-eater	−0.124	0.162	0.883	0.013	0.182	1.013
High meat-eater (reference)						
**Gender**						
Male	**0.405**	**0.137**	1.499**	0.303	0.158	1.354
Female (reference)						
*Age (years, continuous)*	−0.006	0.005	0.994	0.002	0.006	1.002
**Education**						
Low	0.282	0.176	1.326	−0.083	0.200	0.920
Middle	0.148	0.171	1.160	−0.012	0.192	0.988
High (reference)						
**Attitudinal and behavioral variables (factors, continuous)**						
Infrequent cooking/prepared ingredients				0.130	0.079	1.139
Frequent cooking/fresh ingredients				**0.167**	**0.082**	1.182*
Meat attachment				**0.369**	**0.091**	1.446***
Food innovativeness				0.120	0.079	1.127
Dietary guidelines				**−0.579**	**0.096**	0.560***
Sustainable food choice motives				**0.218**	**0.089**	1.244*
Organic food stores				0.125	0.085	0.133
*Nagelkerke R square (%)*	3.6			13.5		

*Significant predictors are displayed in bold font*.

*Est, Estimate; SE, Standard Error; OR, Odds Ratio*.

a *Adjusted for gender, age, and education.*

b *Adjusted for gender, age, education, attitudinal and behavioral variables*.

* *p < 0.05;*

** *p < 0.01;*

*** *p < 0.001*.

## Discussion

This study adds to the discussion on sustainable food consumption by examining the status quo of the consumption of plant-based meat alternatives in Germany together with other UPF groups (i.e., convenience foods, fast foods, snacks, and ultra-processed beverages), gaining a better understanding of the underlying consumption behavior.

### Status Quo of UPF Consumption

The results of this study revealed that the frequency of UPF consumption varies widely along with the examined product groups:

(a) Only a small number of participants (12.3%) reported eating plant-based meat alternatives within the previous 4 weeks
(b) Followed by slightly more than half of the participants (57.4%; 55.9%) who reported consuming convenience products and fast foods
(c) A higher proportion of participants (97.3%; 80.1%) who consumed sweet and salty snacks and ultra-processed beverages.

(a) Concerning plant-based meat alternatives, consumption was rather low and more occasional, with an average eating frequency of two or three times per month. In line with this finding, a recent consumer survey in Germany on the consumption of meat alternatives found a share of 19.3% consumers reported consuming meat substitutes, and within, most (62.2%) are occasional users with a consumption frequency of once a month or less (59). In fact, despite the growing consumer demand for meat alternatives, the use of these products in the daily diet of consumers remains low in Germany. Also, they have still considered niche products in other European countries (60–63). Several studies are currently looking at the perception and acceptance of meat alternatives in order to assist in achieving meat reduction (63–67). However, most of these studies so far do not consider the degree of processing of the products. Meat alternatives should be differentiated according to their degree of processing, from traditional, less processed vegetable protein products, such as legumes and tofu, to highly processed products, whose purpose is to imitate meat and meat products. Further, it is important to clarify whether consumers are aware of the processing behind plant-based meat alternatives and what role such products might play in the diet. Efforts should be made to provide consumer education on preparing meat alternatives (e.g., cooking kits), convenient but minimally processed, and by that, making legumes and other plant-based foods a familiar choice.

(b) Consumption of convenience and fast food products tends to be less frequent, averaging two to three times per month. However, since more than half of consumers reported consuming these products, they are already more entrenched in consumption habits, regardless of frequency. Previous studies on fast food consumption showed, especially among adolescents and students in Germany, a higher consumption, i.e., weekly to even daily (68, 69). In the present study, the average consumption was lower; however, the consumption also appeared strongly correlated with younger age.

(c) Sweet and salty snacks and ultra-processed beverages were most often consumed along UPFs. They were also consumed relatively frequently, averaging one to several times per week. With regard to ultra-processed beverages, there is concern that the accompanying sugar intake -in the form of sugar-sweetened beverages- increases overall energy intake and may reduce the intake of foods containing more nutritionally adequate calories, leading to an unhealthy diet and diet-related diseases (70). In 2016, Germany's per capita consumption of sugar-sweetened beverages was almost equal to that of mineral water (71). Also, in this study, about 30% of participants reported daily consumption of ultra-processed beverages.

Concerning salty snacks, higher consumption may lead to increased salt intake. However, the potential contribution of salty snacks to daily salt intake depends on the average salt content of the different snack types and the amount consumed. Although reports highlight that large parts of the German population consume too much sodium (72), the contribution of snacks to salt intake is unclear. Overall, there is a trend toward more snacks (snackification) in many countries (73).

### UPF Consumption Among Dietary Patterns

The results of this study clearly show that consumption of UPFs differs along with the dietary groups. Plant-based meat alternatives were strongly represented in a vegetarian diet. Several studies described vegetarian diets as beneficial for the environment and also for health because they have higher nutrient quality (11). However, the present analyses demonstrated that vegetarians frequently consume processed meat alternatives, while omnivorous dietary patterns, in contrast, were consistently characterized by low consumption of plant-based meat alternatives. Since industrially produced plant-based meat alternatives are associated with some adverse health (15–17) and sometimes also environmental (29, 34, 35) outcomes as a result of ultra-processing, consumption of such products can be expected to have potential impacts on a plant-based diet. Moreover, it can be assumed that novel protein products might not reduce the demand for animal proteins but lead to an expansion of the market for protein-rich foods, which, however, would not be a desirable development from a sustainability perspective (98). Nutrition policies and dietary guidelines should continue to emphasize a diet of plant-based foods such as nuts, seeds, and legumes, which are rich in protein and many other nutrients but less industrially processed. Even though the technology behind the processes is constantly improving, facing the growing market, it is important to improve current meat substitutes, e.g., nutrients and resource use (60, 75).

Meat-based dietary patterns were increasingly characterized by consuming convenience foods, fast foods, snacks, and ultra-processed beverages. Existing evidence already points to the association between inappropriate eating habits, including inferior cooking behavior, under-/overeating, consuming too many types of ultra-processed foods and drinks, and higher meat consumption (76–78). Although consumption averaged only two to three times per month, the association with a meat-heavy diet suggests that UPF consumption may be embedded in an overall less healthy lifestyle. It stands to reason that a holistic approach to healthy and sustainable diets will require action by different stakeholders across different temporal and spatial scales through different entry points of the food system. Possible population-wide strategies in this regard include fiscal and pricing measures, challenges to defaults and norms of information, and consumer-focused education, more appropriate food labeling, and restrictions on advertising and promotion (79).

A striking result is that flexitarians overall predominantly show significantly lower UPF consumption than meat-eaters and, in some cases, even vegetarians. Concerning meat alternatives, this is consistent with other studies in which moderate meat-eaters who are willing to substitute meat were not found to have increased consumption of meat alternatives (65, 66). Sensory or health reasons are often cited in this regard (80, 81). In general, studies looking at motivations, promoters, and/or barriers to meat reduction indicate that egoistic factors such as taste, health, and nutrition motivate meat reduction more often than prosocial/ethical factors (82–84). It might stand to reason that just as flexitarians do not exhibit increased consumption of plant-based meat alternatives for taste reasons, they also consume less UPF for health reasons. Nevertheless, based on this result, a flexitarian diet can be considered beneficial for sustainability due to lower UPF consumption.

### Factors Associated With UPF Consumption

Variables related to dietary patterns, sociodemographic, attitudes and behaviors toward cooking and consumption were analyzed to predict consumption of ultra-processed foods.

The results of the present study showed that dietary patterns were associated with the consumption of plant-based meat alternatives, fast foods, sweet and salty snacks, and ultra-processed beverages after adjusting for sociodemographic correlates. Only for convenience foods did a significant association emerge only for age. However, for all product groups, but especially for plant-based meat alternatives and highly processed beverages, adjustment for attitudinal and behavioral variables proved to be significant predictors.

The results pointed out cooking behavior as a critical predictor among all ultra-processed product groups, with less frequent cooking and use of mainly instant ingredients significantly related to UPF consumption. Eating sustainable and healthy foods at home requires both time and a certain level of comfort in food preparation (23, 85). However, convenience products, fast food, and even processed meat alternatives are helping to create a culture where time to prepare food is scarce (or perceived to be scarce), and the use of convenience products that require less time, energy, and cooking skills is ubiquitous (23, 24, 86). The shift in consumer demand away from highly processed ready-to-eat or ready-to-heat convenience products toward fresh or home-prepared ingredients requires cooking skills and knowledge to be integrated and taught in nutrition education, and by that, creating some familiarity with preparing plant-based meals (87, 88).

In relation to plant-based meat alternatives, positive attitudes toward meat were found to be significantly negatively associated with the consumption of plant-based meat substitutes. This result is not consistent with the findings of Circus and Robison (89). They found that the personal willingness to consume alternative proteins, in this case, lab-grown meat, edible insects, and plant-based substitutes, was significantly and particularly associated with the attachment to meat. Conversely, however, it is to some extent consistent with a study by Profeta et al. (74), who showed that the more attached consumers were to meat, the less they preferred meat blended with plant-based proteins. They tested products in which only a fraction of the meat (e.g., 20 to 50%) was replaced with plant proteins, reinforcing the assumption that attachment to meat as a psychological construct is a barrier to dietary change. Nonetheless, it stands to reason that the negative association could be explained by the relatively high proportion of vegetarians in the consumption frequency, as vegetarians generally have lower levels of agreement in terms of meat consumption, meat-eating habits, and belief in human supremacy (49, 90). It further appeared that plant-based meat alternative consumption was associated with some adherence to dietary guidelines for healthy eating. That suggests that plant-based meat alternative consumers value healthy eating even though meat substitutes are highly processed foods. It is possible that the level of processing is either not perceived as such or is not associated with health and thus is not a barrier to plant-based meat alternative consumption.

An intriguing finding concerns the negative correlation between plant-based meat alternative consumption and motivations for sustainable food choices. Indeed, the evidence on plant-based meat alternative acceptance is mixed, and the analysis on drivers of consumption remains inconsistent (60, 91). Some authors found that consumers with perceptions regarding the high environmental impact of meat were more likely to consume meat substitutes when compared with people who had the opposite attribute (62, 92). Close to this, Michel et al. (63) found that non-meat eaters perceived meat alternatives to be better in terms of environmental friendliness. However, the authors did not differentiate the processing levels of meat alternatives in the survey by integrating lentils and tofu as well as meat substitutes, which limits the transferability of their conclusion. Besides, some other work suggests that the sustainability motive in food decisions does not play an integral, consistent role in accepting alternative protein sources (64). Moreover, alternative attributes and/or attributes related to meat, such as sensory attributes, are considered important factors influencing purchase intentions, rather than environmental considerations, convenience, or healthy purchase decisions (93, 94). Consistent with this, it would be possible that motives not included in this study, such as taste, price, and convenience, strongly influence the identified frequency of consumption of plant-based meat alternatives, thus biasing the analysis to some extent. However, this statement would need to be investigated further.

Finally, there was a significant relationship between consumption of plant-based meat alternatives and shopping at organic food markets and regional stores. These results suggest that the market and availability for UPF are growing in the area of more environmentally sustainable consumption. However, this may mask a conflict of interest. Organic markets aim to strengthen and promote sustainable food systems and supply chains (95) and may underestimate the highly processed nature of these products, which conflicts with overall nutrition and health goals.

Within sociodemographic correlations, age was a strong predictor for all ultra-processed product groups, decreasing age predicted increased consumption. This finding is in line with previous studies, as younger people tend to consume more food out of home, at work and spend less time in cooking, while older people are associated with spending more time in cooking and are less familiar with convenience products (21, 24). It was also shown that women were more likely to be high consumers of plant-based meat alternatives and sweet and salty snacks, but not ultra-processed beverages, compared with men. These findings are supported to some extent by previous studies showing increased consumption of sugar-sweetened soft drinks and processed meats and lower consumption of sweets and plant-based meat alternatives among men compared to women (38, 62, 96). In addition, the analyses revealed that the likelihood of consuming plant-based meat alternatives increased with a higher level of education. That is consistent with previous studies, in which people of a higher level of education are thought to be more aware of the health and/or environmental benefits of a predominantly plant-based diet (64, 97).

### Merits and Limitations

Significant merit of the current study was the use of separate scores indicative of UPF consumption (plant-based meat alternatives, convenience foods, fast foods, sweet and salty snacks, and ultra-processed beverages), as it is reasonable to assume that different factors are influencing the consumption of plant-based meat alternatives and conventional UPF. Another strength was using an accurate meat consumption score to form dietary patterns. To the best of our knowledge, no study in Germany has distinguished high-meat eaters, consuming much more meat than recommended (> 150 g/d), even though this share of consumption is strongly represented in Germany. With this, the current study could make a valuable contribution to the development of research that works with or examines meat consumption.

Nevertheless, there were also some limitations of the study. In terms of sociodemographic databases, the study sample overrepresented women, various age groups, and participants with middle and lower levels of education. Regarding the frequency measure of UPF consumption, it is possible that a more detailed query regarding individual items would have been more appropriate. In this respect, the grouping of foods used as indicators of UPF consumption could have been more precise.

The hypotheses and design of the study as a whole were only conducted under the ethical principles of the German Psychological Society (DGP) and the Professional Association of German Psychologists (BDP), but were not additionally pre-registered as a study by any particular journal or the international scientific community; this is an indication of future endeavors. However, the entire approach and design were financed with public funds, involving a two-step funding procedure and a public rehearsal. The study was defended and reviewed by several external scientific experts.

## Conclusion

As the proportion of ultra-high processed products is steadily increasing, not yet considering the growing share of plant-based meat alternatives, and a broad body of studies points to the association with diet-related diseases, it is crucial to constantly investigate the proportion of such products in society. This study shows that the frequency of consumption of UPF varies significantly not only across the product groups studied but also in relation to different dietary patterns. While consumption of plant-based meat alternatives predominated in vegetarian diets, convenience products, fast foods, snacks, and ultra-processed beverages were strongly associated with meat-rich diets. Strikingly, flexitarians have low consumption of all types of ultra-processed foods, which is an important finding for sustainability assessment of this dietary pattern. Previous research has done much to understand meat substitute acceptance, motivations, and barriers behind consumption, but mostly in the context of meat reduction. Indeed, plant-based meat alternatives may have some beneficial aspects compared to meat, but there is no evidence that they can replace a healthy diet focused on minimally processed plant foods, as proposed by the Planetary Health Diet (10). Future studies should examine consumer awareness and attitudes to gain a more comprehensive understanding of the perception of meat alternatives as ultra-processed foods to address the unprecedented challenge of healthy and sustainable diets. Also, it is crucial that future research targets in-depth investigations, for example, on the nutritional quality of plant-based alternative-oriented diets, with high content of processed products. Dietary policies and guidelines should further emphasize diets rich in plant foods such as nuts, seeds, and legumes that are high in protein and many other nutrients and less industrially processed. As cooking behaviors were a significant predictor of UPF consumption, it is critical that education, increased nutrition literacy, and ultimately a range of policies and interventions aim to create a culture in which healthy and sustainable diets are being practiced, accessible and feasible for the broader community.

## Data Availability Statement

The raw data supporting the conclusions of this article will be made available by the authors, without undue reservation.

## Author Contributions

AR and MO conceptualized the experiment and ran the experiment. MO conducted data analysis and supervised by AR. AS, MO, and AR contributed to the interpretation of the results. MO wrote the manuscript. AR and AS revised the manuscript. All authors gave final approval for the submitted manuscript.

## Funding

The work was funded by Volkswagen Foundation, grant number ZN3382, and Ministry for Science and Culture of Lower Saxony (MWK), grant number VWZN3255. The authors are responsible for the content of this publication.

## Conflict of Interest

The authors declare that the research was conducted in the absence of any commercial or financial relationships that could be construed as a potential conflict of interest.

## Publisher's Note

All claims expressed in this article are solely those of the authors and do not necessarily represent those of their affiliated organizations, or those of the publisher, the editors and the reviewers. Any product that may be evaluated in this article, or claim that may be made by its manufacturer, is not guaranteed or endorsed by the publisher.

## Footnotes

^1^ https://www.fao.org/faoterm/viewentry/en/?entryId=175467

^2^ https://www.dge.de/fileadmin/public/doc/en/10-guidelines-wholesome-diet-dge.pdf
