# Supplementary material for: Plant-Based Diets Are Not Enough? Understanding the Consumption of Plant-Based Meat Alternatives Along Ultra-processed Foods in Different Dietary Patterns in Germany
Source: Front Nutr. 2022 Apr 27;9:852936. doi: 10.3389/fnut.2022.852936 (PMC9094491; doi:10.3389/fnut.2022.852936)
Supplement: Supplementary file 1 [file Data_Sheet_1.docx]

Supplementary Material

# Appendix A Supplementary Data

Table A1: Factor loadings from principal axis factor analysis

|  | **Factor** | Frequent cooking/fresh ingredients | Infrequent cooking/prepared ingredients | Meat attachment | Food innovativeness | Dietary guidelines | Food choice motives (sustainability) | Food choice motives (other) | Conventional grocery stores | Organic grocery stores |
| --- | --- | --- | --- | --- | --- | --- | --- | --- | --- | --- |
| Average time I spend preparing a main meal in a day during the week (apart from the breakfast)^1^ | | 0.810 |  |  |  |  |  |  |  |  |
| Average time I spend preparing a main meal in a day during the weekend (apart from the breakfast)^1^ | | 0.805 |  |  |  |  |  |  |  |  |
| Frequency on how often I cook myself a hot meal^2^ | | 0.614 |  |  |  |  |  |  |  |  |
| The ingredients I use for cooking are mainly fresh and unprocessed^2^ | | 0.610 |  |  |  |  |  |  |  |  |
| The ingredients I use for cooking are completely prepared (e.g. frozen pizza)^2^ | |  | 0.727 |  |  |  |  |  |  |  |
| The ingredients I use for cooking are mainly ready prepared (e.g. instant sauces)^2^ | |  | 0.851 |  |  |  |  |  |  |  |
| The ingredients I use for cooking are half-finished / half-fresh and unprocessed^2^ | |  | 0.811 |  |  |  |  |  |  |  |
| *How much do you agree with the following statements^3^* | |  |  |  |  |  |  |  |  |  |
|  | Eating meat is not that harmful. |  |  | 0.779 |  |  |  |  |  |  |
|  | Meat gives me strength. |  |  | 0.785 |  |  |  |  |  |  |
|  | Eating meat is important to my family and friends. |  |  | 0.524 |  |  |  |  |  |  |
|  | It is "natural" to eat meat because it is human nature. |  |  | 0.834 |  |  |  |  |  |  |
|  | I like to eat high quality meat. |  |  | 0.463 |  |  |  |  |  |  |
|  | Meat is my vegetable. |  |  | 0.650 |  |  |  |  |  |  |
|  | It is a pleasure to eat meat because it is part of many good tasting dishes. |  |  | 0.811 |  |  |  |  |  |  |
|  | It is "necessary" to eat meat because it contains important nutrients, such as vitamin B12 and iron. |  |  | 0.812 |  |  |  |  |  |  |
|  | It is "normal" to eat meat because most people eat meat. |  |  | 0.759 |  |  |  |  |  |  |
|  | Vegan diet is unhealthy |  |  | 0.569 |  |  |  |  |  |  |
|  | Vegans are crazy weirdos |  |  | 0.649 |  |  |  |  |  |  |
|  | I like to try new and unknown foods |  |  |  | 0.895 |  |  |  |  |  |
|  | I like the food of other cultures. |  |  |  | 0.823 |  |  |  |  |  |
|  | I like to try new food trends. |  |  |  | 0.678 |  |  |  |  |  |
|  | I prefer to eat dishes that I am familiar with or used to. |  |  |  | -0.761 |  |  |  |  |  |
|  | I eat 5 servings of fruits and vegetables a day. |  |  |  |  | 0.551 |  |  |  |  |
|  | I consciously watch my salt consumption. |  |  |  |  | 0.747 |  |  |  |  |
|  | I consciously watch my sugar consumption. |  |  |  |  | 0.731 |  |  |  |  |
|  | I make sure I eat enough fats that are good for my health. |  |  |  |  | 0.797 |  |  |  |  |
|  | I make sure to drink enough water. |  |  |  |  | 0.608 |  |  |  |  |
|  | I try to eat as varied a diet as possible. |  |  |  |  | 0.737 |  |  |  |  |
|  | I take time to eat. |  |  |  |  | 0.531 |  |  |  |  |
|  | I prefer whole grain products to simple wheat flour products. |  |  |  |  | 0.597 |  |  |  |  |
| *How important are the following food quality characteristics to you?^4^* | |  |  |  |  |  |  |  |  |  |
|  | Regionality |  |  |  |  |  | 0.790 |  |  |  |
|  | Animal welfare |  |  |  |  |  | 0.842 |  |  |  |
|  | Animal husbandry |  |  |  |  |  | 0.850 |  |  |  |
|  | Seasonality |  |  |  |  |  | 0.734 |  |  |  |
|  | fair trade |  |  |  |  |  | 0.831 |  |  |  |
|  | Organic production |  |  |  |  |  | 0.807 |  |  |  |
|  | Transport |  |  |  |  |  | 0.819 |  |  |  |
|  | Naturalness |  |  |  |  |  | 0.702 |  |  |  |
|  | Without genetic engineering |  |  |  |  |  | 0.668 |  |  |  |
|  | DLG quality test |  |  |  |  |  | 0.699 |  |  |  |
|  | Freshness* |  |  |  |  |  |  | 0.816 |  |  |
|  | Taste* |  |  |  |  |  |  | 0.853 |  |  |
|  | Price* |  |  |  |  |  |  | 0.526 |  |  |
| Where do you buy your food?^2^ | |  |  |  |  |  |  |  |  |  |
|  | Delivery service* |  |  |  |  |  |  |  | 0.476 |  |
|  | Discounter* |  |  |  |  |  |  |  | -0.710 |  |
|  | Supermarket* |  |  |  |  |  |  |  | 0.527 |  |
|  | Weekly market |  |  |  |  |  |  |  |  | 0.740 |
|  | Organic supermarket |  |  |  |  |  |  |  |  | 0.776 |
|  | Farmer, direct marketer |  |  |  |  |  |  |  |  | 0.741 |
|  | Health food stores |  |  |  |  |  |  |  |  | 0.710 |
|  | Private production |  |  |  |  |  |  |  |  | 0.623 |
|  | N | 791 | 791 | 791 | 811 | 796 | 773 | 773 | 775 | 775 |
|  | Mean of construct | 3.51 | 2.31 | 3.20 | 3.20 | 3.17 | 3.62 | 4.52 | 3.12 | 1.77 |
|  | Standard deviation of construct | 0.959 | 0.942 | 1.157 | 1.064 | 1.137 | 1.160 | 0.754 | 0.879 | 0.970 |
|  | Cronbach’s alpha | 0.76 | 0.74 | 0.90 | 0.74 | 0.82 | 0.95 | 0.53 | 0.13 | 0.75 |

^1^ 5-point-scale: Less than 15 minutes; between 16 - 30 minutes; between 31 - 45 minutes; between 46 - 60 minutes; bore than 60 minutes.

^2^ 5-point-scale: Never, rarely, sometimes, often, very often

^3^ 5-point-scale: Strongly disagree, disagree, neutral/uncertain, agree, strongly agree

^4^ 5-point-scale: Very unimportant, unimportant, neutral/uncertain, important, very important

* Items were removed as they did not have a reliable scale

Table A2: Results of a binary logistic regression analysis predicting consumption of plant-based meat alternatives (N= 814). Significant predictors are displayed in bold font.

|  |  | Unadjusted | | | Adjusted | | | | | |
| --- | --- | --- | --- | --- | --- | --- | --- | --- | --- | --- |
|  |  | *B* | SE | p | *B*^a^ | SE | p | *B*^b^ | SE | p |
| *Diet* | |  |  |  |  |  |  |  |  |  |
|  | Vegetarian | **2.251** | **0.397** | **0.000** | **2.008** | **0.414** | **0.000** | 0.248 | 0.565 | 0.660 |
|  | Flexitarian | 0.600 | 0.374 | 0.108 | 0.459 | 0.386 | 0.235 | 0.189 | 0.442 | 0.668 |
|  | Regular meat- eaters | 0.634 | 0.350 | 0.070 | 0.456 | 0.363 | 0.208 | 0.372 | 0.405 | 0.359 |
|  | High meat- eaters (reference) |  |  |  |  |  |  |  |  |  |
| *Gender* | |  |  |  |  |  |  |  |  |  |
|  | Male |  |  |  | **-0.668** | **0.288** | **0.020** | -0.336 | 0,335 | 0.315 |
|  | Female (reference) |  |  |  |  |  |  |  |  |  |
| *Age (years, continuous)* | |  |  |  | **-0.029** | **0.010** | **0.002** | **-0.037** | **0,012** | **0.002** |
| *Education* | |  |  |  |  |  |  |  |  |  |
|  | Low |  |  |  | **-0.797** | **0.367** | **0.030** | -0.108 | 0,433 | 0.803 |
|  | Middle |  |  |  | -0.278 | 0.298 | 0.349 | 0.405 | 0,348 | 0.245 |
|  | High (reference) |  |  |  |  |  |  |  |  |  |
| *Attitudinal and behavioral variables (factors, continuous)* | |  |  |  |  |  |  |  |  |  |
|  | Infrequent cooking/prepared ingredients |  |  |  |  |  |  | **0.376** | **0,163** | **0.021** |
|  | Frequent cooking/fresh ingredients |  |  |  |  |  |  | -0.227 | 0,176 | 0.198 |
|  | Meat attachment |  |  |  |  |  |  | **-1.191** | **0,204** | **0.000** |
|  | Food innovation |  |  |  |  |  |  | **0.475** | **0,168** | **0.005** |
|  | Dietary guidelines |  |  |  |  |  |  | **0.416** | **0,194** | **0.032** |
|  | Sustainable food choices |  |  |  |  |  |  | **-0.406** | **0,193** | **0.036** |
|  | Organic food stores |  |  |  |  |  |  | **0.562** | **0,155** | **0.000** |
| Nagelkerke R square (%) | | 9.9 |  |  | 19.0 |  |  | 42.0 |  |  |

^a^ Adjusted for gender, age, and education. ^b^ Adjusted for gender, age, education, attitudinal and behavioral variables.

Table A3: Results of a binary logistic regression analysis predicting convenience food consumption (N= 814). Significant predictors are displayed in bold font.

|  |  | Unadjusted | | | Adjusted | | | | | |
| --- | --- | --- | --- | --- | --- | --- | --- | --- | --- | --- |
|  |  | Beta | SE | p | *B*^a^ | SE | p | *B*^b^ | SE | p |
| *Diet* | |  |  |  |  |  |  |  |  |  |
|  | Vegetarian | -0.367 | 0.317 | 0.247 | -0.512 | 0.325 | 0.115 | -0.324 | 0.407 | 0.426 |
|  | Flexitarian | -0.352 | 0.219 | 0.107 | -0.395 | 0.224 | 0.078 | -0.278 | 0.248 | 0.261 |
|  | Regular meat-eaters | -0.210 | 0.203 | 0.300 | -0.259 | 0.208 | 0.212 | -0.094 | 0.227 | 0.678 |
|  | High meat-eaters (reference) |  |  |  |  |  |  |  |  |  |
| *Gender* | |  |  |  |  |  |  |  |  |  |
|  | Male |  |  |  | -0.117 | 0.172 | 0.496 | -0.199 | 0.196 | 0.309 |
|  | Female (reference) |  |  |  |  |  |  |  |  |  |
| *Age (years, continuous)* | |  |  |  | **-0.023** | **0.006** | **0.000** | -0.008 | 0.007 | 0.270 |
| *Education* | |  |  |  |  |  |  |  |  |  |
|  | Low |  |  |  | 0.196 | 0.221 | 0.374 | 0.075 | 0.250 | 0.765 |
|  | Middle |  |  |  | -0.006 | 0.216 | 0.977 | -0.068 | 0.240 | 0.776 |
|  | High (reference) |  |  |  |  |  |  |  |  |  |
| *Attitudinal and behavioral variables (factors, continuous)* | |  |  |  |  |  |  |  |  |  |
|  | Infrequent cooking/prepared ingredients |  |  |  |  |  |  | **0,950** | **0.114** | **0.000** |
|  | Frequent cooking/fresh ingredients |  |  |  |  |  |  | 0.010 | 0.101 | 0.918 |
|  | Meat attachment |  |  |  |  |  |  | -0.128 | 0.112 | 0.256 |
|  | Food innovation |  |  |  |  |  |  | 0.019 | 0.097 | 0.841 |
|  | Dietary guidelines |  |  |  |  |  |  | -0.107 | 0.117 | 0.358 |
|  | Sustainable food choices |  |  |  |  |  |  | -0.058 | 0.110 | 0.602 |
|  | Organic food stores |  |  |  |  |  |  | 0.143 | 0.106 | 0.178 |
| Nagelkerke R square (%) | | 0.7 |  |  | 4.0 |  |  | 22.9 |  |  |

^a^ Adjusted for gender, age, and education. ^b^ Adjusted for gender, age, education, attitudinal and behavioral variables.

Table A4: Results of a binary logistic regression analysis predicting fast food consumption (N= 814). Significant predictors are displayed in bold font.

|  |  | Unadjusted | | | Adjusted | | | | | |
| --- | --- | --- | --- | --- | --- | --- | --- | --- | --- | --- |
|  |  | Beta | SE | p | *B*^a^ | S | p | *B*^b^ | SE | p |
| *Diet* | |  |  |  |  |  |  |  |  |  |
|  | Vegetarian | -0.400 | 0.315 | 0.204 | **-0.889** | **0.345** | **0.010** | **-0.961** | **0.401** | **0.017** |
|  | Flexitarian | -0.308 | 0.216 | 0.155 | -0.397 | 0.236 | 0.093 | -0.374 | 0.249 | 0.132 |
|  | Regular meat-eaters | 0.107 | 0.203 | 0.599 | 0.000 | 0.219 | 0.999 | 0.068 | 0.227 | 0.763 |
|  | High meat-eaters (reference) |  |  |  |  |  |  |  |  |  |
| *Gender* | |  |  |  |  |  |  |  |  |  |
|  | Male |  |  |  | 0.206 | 0.182 | 0.257 | 0.175 | 0.197 | 0.376 |
|  | Female (reference) |  |  |  |  |  |  |  |  |  |
| *Age (years, continuous)* | |  |  |  | **-0.050** | **0.007** | **0.000** | **-0.044** | **0.007** | **0.000** |
| *Education* | |  |  |  |  |  |  |  |  |  |
|  | Low |  |  |  | -0.342 | 0.230 | 0.136 | -0.263 | 0.246 | 0.286 |
|  | Middle |  |  |  | -0.131 | 0.233 | 0.573 | -0.076 | 0.244 | 0.755 |
|  | High (reference) |  |  |  |  |  |  |  |  |  |
| *Attitudinal and behavioral variables (factors, continuous)* | |  |  |  |  |  |  |  |  |  |
|  | Infrequent cooking/prepared ingredients |  |  |  |  |  |  | **0.356** | **0.099** | **0.000** |
|  | Frequent cooking/fresh ingredients |  |  |  |  |  |  | -0.111 | 0.101 | 0.272 |
|  | Meat attachment |  |  |  |  |  |  | -0.081 | 0.113 | 0.474 |
|  | Food innovation |  |  |  |  |  |  | 0.137 | 0.097 | 0.161 |
|  | Dietary guidelines |  |  |  |  |  |  | -0.082 | 0.115 | 0.477 |
|  | Sustainable food choices |  |  |  |  |  |  | -0.111 | 0.110 | 0.312 |
|  | Organic food stores |  |  |  |  |  |  | **0.277** | **0.107** | **0.010** |
| Nagelkerke R square (%) | | 1.2 |  |  | 18.7 |  |  | 23.1 |  |  |

^a^ Adjusted for gender, age, and education. ^b^ Adjusted for gender, age, education, attitudinal and behavioral variables.

Table A5: Results of an ordinal regression analysis predicting sweet and salty snack consumption (N= 814). Significant predictors are displayed in bold font.

|  |  | Unadjusted | | | Adjusted | | | | | |
| --- | --- | --- | --- | --- | --- | --- | --- | --- | --- | --- |
|  |  | Est | SE | p | Est^a^ | SE | p | Est^b^ | SE | p |
| *Diet* | |  |  |  |  |  |  |  |  |  |
|  | Vegetarian | **-0.497** | **0.247** | **0.044** | **-0.699** | **0.263** | **0.008** | -0.439 | 0.323 | 0.174 |
|  | Flexitarian | **-0.469** | **0.168** | **0.005** | **-0.531** | **0.179** | **0.003** | -0.360 | 0.199 | 0.070 |
|  | Regular meat-eaters | -0.248 | 0.150 | 0.099 | -0.258 | 0.162 | 0.110 | -0.141 | 0.180 | 0.432 |
|  | High meat- eaters (reference) |  |  |  |  |  |  |  |  |  |
| *Gender* | |  |  |  |  |  |  |  |  |  |
|  | Male |  |  |  | **-0.267** | **0.136** | **0.013** | **-0.379** | **0.157** | **0.016** |
|  | Female (reference) |  |  |  |  |  |  |  |  |  |
| *Age (years, continuous)* | |  |  |  | **-0.016** | **0.005** | **0.000** | -0.005 | 0.006 | 0.393 |
| *Education* | |  |  |  |  |  |  |  |  |  |
|  | Low |  |  |  | 0.005 | 0.175 | 0.977 | -0.224 | 0.198 | 0.258 |
|  | Middle |  |  |  | -0.207 | 0.171 | 0.225 | -0.252 | 0.191 | 0.186 |
|  | High (reference) |  |  |  |  |  |  |  |  |  |
| *Attitudinal and behavioral variables (factors, continuous)* | |  |  |  |  |  |  |  |  |  |
|  | Infrequent cooking/ prepared ingredients |  |  |  |  |  |  | **0.244** | **0.078** | **0.002** |
|  | Frequent cooking/ fresh ingredients |  |  |  |  |  |  | 0.132 | 0.081 | 0.103 |
|  | Meat attachment |  |  |  |  |  |  | 0.010 | 0.089 | 0.912 |
|  | Food innovation |  |  |  |  |  |  | 0.116 | 0.078 | 0.135 |
|  | Dietary guidelines |  |  |  |  |  |  | **-0.386** | **0.093** | **0.000** |
|  | Food choice motives |  |  |  |  |  |  | 0.089 | 0.088 | 0.311 |
|  | Organic food stores |  |  |  |  |  |  | 0.081 | 0.084 | 0.334 |
| Nagelkerke R square (%) | | 1.2 |  |  | 3.6 |  |  | 8.9 |  |  |

^a^ Adjusted for gender, age, and education. ^b^ Adjusted for gender, age, education, attitudinal and behavioral variables.

TableA6: Results of an ordinal regression analysis predicting ultra-processed beverage consumption (N= 814). Significant predictors are displayed in bold font.

|  |  | Unadjusted | | | Adjusted | | | | | |
| --- | --- | --- | --- | --- | --- | --- | --- | --- | --- | --- |
|  |  | Est | SE | p | Est^a^ | SE | p | Est^b^ | SE | p |
| *Diet* | |  |  |  |  |  |  |  |  |  |
|  | Vegetarian | **-0.986** | **0.249** | **0.000** | **-0.883** | **0.264** | **0.001** | -0.268 | 0.329 | 0.415 |
|  | Flexitarian | **-0.429** | **0.168** | **0.011** | -0.278 | 0.179 | 0.120 | -0.076 | 0.200 | 0.704 |
|  | Regular meat- eaters | -0.157 | 0.151 | 0.300 | -0.124 | 0.162 | 0.444 | 0.013 | 0.182 | 0.942 |
|  | High meat- eaters (reference) |  |  |  |  |  |  |  |  |  |
| *Gender* | |  |  |  |  |  |  |  |  |  |
|  | Male |  |  |  | **0.405** | **0.137** | **0.003** | 0.303 | 0.158 | 0.055 |
|  | Female (reference) |  |  |  |  |  |  |  |  |  |
| *Age (years, continuous)* | |  |  |  | -0.006 | 0.005 | 0.194 | 0.002 | 0.006 | 0.697 |
| *Education* | |  |  |  |  |  |  |  |  |  |
|  | Low |  |  |  | 0.282 | 0.176 | 0.109 | -0.083 | 0.200 | 0.679 |
|  | Middle |  |  |  | 0.148 | 0.171 | 0.386 | -0.012 | 0.192 | 0.949 |
|  | High (reference) |  |  |  |  |  |  |  |  |  |
| *Attitudinal and behavioral variables (factors, continuous)* | |  |  |  |  |  |  |  |  |  |
|  | Infrequent cooking/ prepared ingredients |  |  |  |  |  |  | 0.130 | 0.079 | 0.102 |
|  | Frequent cooking/ fresh ingredients |  |  |  |  |  |  | **0.167** | **0.082** | **0.042** |
|  | Meat attachment |  |  |  |  |  |  | **0.369** | **0.091** | **0.000** |
|  | Food innovation |  |  |  |  |  |  | 0.120 | 0.079 | 0.129 |
|  | Dietary guidelines |  |  |  |  |  |  | **-0.579** | **0.096** | **0.000** |
|  | Food choice motives |  |  |  |  |  |  | **0.218** | **0.089** | **0.015** |
|  | Organic food stores |  |  |  |  |  |  | 0.125 | 0.085 | 0.139 |
| Nagelkerke R square (%) | | 2.2 |  |  | 3.6 |  |  | 13.5 |  |  |

^a^ Adjusted for sex, age, and education. ^b^ Adjusted for sex, age, education, attitudinal and behavioral variables.
